# Concurrent Lupus Nephritis and Lupus Carditis in a Young Woman With Systemic Lupus Erythematosus: A Case Report With an Incidental Arachnoid Cyst

**DOI:** 10.7759/cureus.94138

**Published:** 2025-10-08

**Authors:** Ahmad Mohammad, Bhavna Singla, Shivam Singla, Sahil Kumar, Arif Tajammul

**Affiliations:** 1 Internal Medicine, Hurley Medical Center, Flint, USA; 2 Internal Medicine, Erie County Medical Center Health Campus, Buffalo, USA; 3 Internal Medicine, TidalHealth Peninsula Regional, Salisbury, USA; 4 Internal Medicine, Liaquat National Hospital, Karachi, PAK; 5 Internal Medicine, Services Hospital Lahore, Lahore, PAK

**Keywords:** arachnoid cyst, autoimmune disease, case report, lupus carditis, lupus nephritis, multi-organ involvement, systemic lupus erythematosus

## Abstract

Systemic lupus erythematosus (SLE) is a complex autoimmune disorder with highly variable clinical presentations. We report the case of a 32-year-old woman who presented with fever, polyarthritis, rash, and renal and cardiac involvement. Laboratory and immunological investigations confirmed SLE with Class IV lupus nephritis and lupus carditis, while brain imaging incidentally revealed an arachnoid cyst. The patient received high-dose corticosteroids, cyclophosphamide, and hydroxychloroquine, resulting in marked clinical and laboratory improvement, with the stabilization of renal function and the resolution of cardiac inflammation. This case highlights the importance of early recognition of severe multi-organ involvement in SLE, the application of standardized diagnostic criteria, and the benefits of a multidisciplinary treatment approach. It also emphasizes the need for careful interpretation of incidental findings to avoid misattribution, providing valuable teaching points for clinicians managing complex autoimmune disease presentations.

## Introduction

Systemic lupus erythematosus (SLE) is a chronic autoimmune disease with a heterogeneous clinical course, ranging from mild mucocutaneous involvement to severe, life-threatening multi-organ damage [1]. Women of reproductive age are disproportionately affected, with a female-to-male ratio of approximately 9:1, and early recognition of organ-threatening complications is essential to improve outcomes, as delayed treatment can lead to irreversible organ damage within six months of onset [2]. Among these, lupus nephritis occurs in up to 40-70% of patients and remains a major cause of morbidity and mortality, while cardiac manifestations such as pericarditis, myocarditis, and valvular disease are reported in 20-30% of cases; though less frequent at initial presentation, they carry significant prognostic implications [2].

The concurrent presentation of severe nephritis and carditis occurs in fewer than 5-10% of newly diagnosed SLE patients and represents a diagnostic and therapeutic challenge requiring a coordinated, multidisciplinary approach. Additionally, incidental neurological findings in patients with SLE can complicate clinical reasoning and demand careful interpretation to avoid misattribution of symptoms [3]. This case report describes a young woman with newly diagnosed SLE presenting with both lupus nephritis and lupus carditis, alongside an incidental arachnoid cyst. The report highlights the importance of applying standardized diagnostic criteria, recognizing rare multi-organ involvement, and implementing timely, aggressive immunosuppressive therapy to prevent irreversible organ damage.

## Case presentation

A 32-year-old married woman presented to our outpatient department with a 2-3-month history of intermittent low-grade fever, progressive polyarthritis, generalized weakness, frothy urine, and photosensitivity. Over the past 15 days, her symptoms had intensified, with worsening shortness of breath, severe right-sided temporal headaches, and marked fatigue interfering with daily activities.

She reported morning stiffness lasting approximately one hour and erythematous rashes over both hands that worsened with sun exposure. There was no prior history of chronic illness, drug use, or smoking. She denied any prescription medications, having taken only occasional homeopathic remedies for joint pain, which was important to note as it ruled out drug-induced lupus. Family history was notable for an unspecified autoimmune disorder in her maternal lineage. She had delivered four healthy children.

On examination, the patient appeared pale and fatigued. Vitals showed the following: temperature 38.2°C, pulse rate 96/min, blood pressure 135/85 mmHg, respiratory rate 20/min, and oxygen saturation 96% on room air. Cutaneous examination revealed erythematous maculopapular rashes on the dorsum of both hands. Cardiovascular examination revealed a pericardial rub, while respiratory examination demonstrated reduced breath sounds in the left lower zone. Musculoskeletal examination showed tenderness and swelling in multiple metacarpophalangeal joints and bilateral knees. Neurological examination was normal, apart from severe headaches reported by the patient. Laboratory investigations revealed multi-organ involvement consistent with active SLE (Table 1).

**Table 1:** Baseline laboratory parameters demonstrating multi-organ involvement in systemic lupus erythematosus. WBC: white blood cell; ESR: erythrocyte sedimentation rate; CRP: C-reactive protein

Parameter	Result	Reference range
Hemoglobin	11.4 g/dL	13-18 g/dL
WBC count	12.3×10⁹/L	4-11×10⁹/L
Platelets	267×10⁹/L	150-400×10⁹/L
ESR	28 mm/hr	0-25 mm/hr
CRP	7.5 mg/L	<5 mg/L
Urea	100 mg/dL	10-50 mg/dL
Creatinine	2.3 mg/dL	0.5-0.9 mg/dL
24-hour urinary protein	5400 mg/24 hr	<140 mg/24 hr

**Figure 1:**
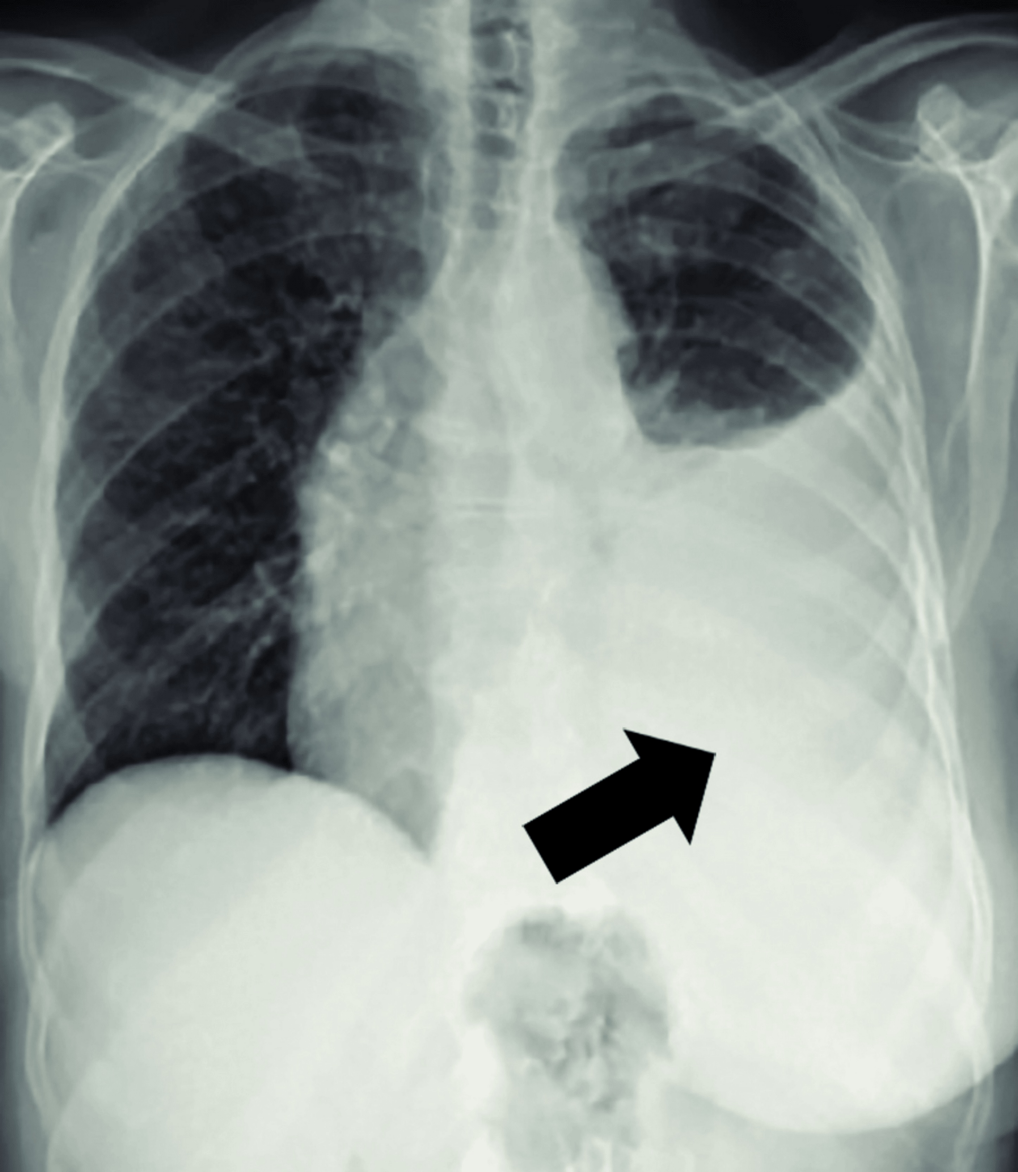
Chest X-ray showing mild left lower lobe pleural effusion (indicated by arrow) consistent with serositis in systemic lupus erythematosus.

**Figure 2:**
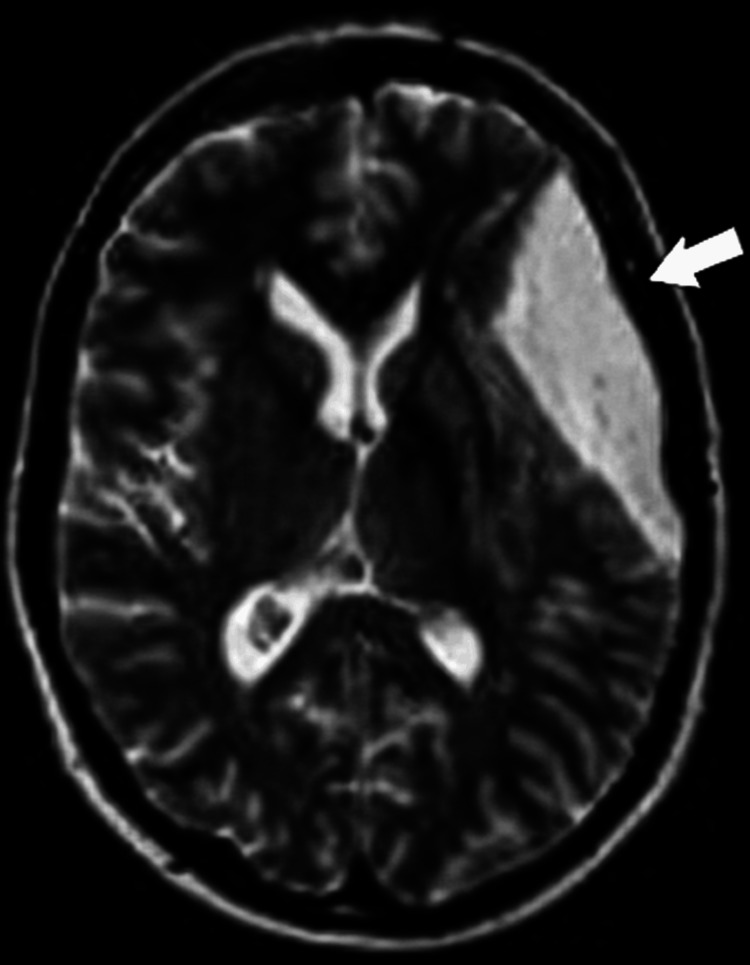
Axial T2-weighted brain MRI showing an incidental arachnoid cyst in the right anterior temporal region (arrow) with mild mass effect on adjacent brain parenchyma. MRI: magnetic resonance imaging

Urinalysis confirmed active urinary sediment with microscopic hematuria (14-16 red blood cells per high-power field) and mild pyuria (5-6 white blood cells per high-power field), supporting the diagnosis of active lupus nephritis alongside the quantitative proteinuria of 5400 mg/24 hr noted in Table 1. Immunological testing revealed strongly positive antinuclear antibodies (homogeneous pattern, titer 1:640) and markedly elevated anti-double-stranded deoxyribonucleic acid (anti-dsDNA) levels (>150 IU/ml), accompanied by reduced complement levels, with C3 measured at 52 mg/dL and C4 at 5 mg/dL. Imaging investigations further supported multi-organ involvement: chest X-ray revealed a mild left lower lobe pleural effusion (Figure 1), while echocardiography showed evidence of mild pericarditis with preserved left ventricular function (ejection fraction 65%) and no signs of cardiac tamponade. Given the patient's persistent severe headaches and concern for possible neuropsychiatric lupus, a brain magnetic resonance imaging (MRI) was performed, which identified an extra-axial cerebrospinal fluid signal intensity lesion in the right anterior temporal region (Figure 2), consistent with an arachnoid cyst causing mild mass effect on adjacent brain parenchyma, with no evidence of hemorrhage or infarction.

Taken together, these findings fulfilled several clinical and immunological criteria of the 2019 European League Against Rheumatism/American College of Rheumatology (EULAR/ACR) classification system, yielding a total score of 37 points, which is substantially higher than the 10-point threshold and reflects severe multi-organ involvement at presentation, as most newly diagnosed patients typically score between 10 and 20 points. The details are provided in Table 2.

**Table 2:** 2019 EULAR/ACR classification criteria scoring for SLE diagnosis. Clinical and laboratory features were scored according to the 2019 EULAR/ACR classification criteria, demonstrating severe multi-organ involvement with a total score of 37 points (threshold ≥10 points for SLE diagnosis). EULAR: European League Against Rheumatism; ACR: American College of Rheumatology; SLE: systemic lupus erythematosus; Anti-dsDNA: anti-double-stranded deoxyribonucleic acid

Domain	Finding	Points
Constitutional	Fever >38.3°C	2
Mucocutaneous	Photosensitive rash	4
Musculoskeletal	Polyarthritis with morning stiffness	6
Serositis	Pericarditis (6), pleural effusion (5)	11
Renal	Proteinuria >0.5 g/day	4
Immunologic	Anti-dsDNA positive	6
Complement	Low C3 and C4	4
Total		37

Based on the clinical presentation, laboratory findings, and imaging results, a diagnosis of SLE with multi-organ involvement was established. The patient fulfilled multiple domains of the 2019 EULAR/ACR classification criteria, with a cumulative score of 37 points, far exceeding the threshold required for classification. The major organ manifestations included Class IV lupus nephritis, suggested by nephrotic-range proteinuria of 5.4 g/24 hr and elevated serum creatinine at 2.3 mg/dL, and lupus carditis, characterized by pericarditis and a left-sided pleural effusion. In addition, a right anterior temporal arachnoid cyst was incidentally identified on brain MRI during the workup of persistent headaches, which was determined to be unrelated to the underlying autoimmune pathology.

The management plan was developed through a multidisciplinary approach involving rheumatology, nephrology, cardiology, and neurology teams. Given the severity of nephritis and cardiac involvement, the patient was started on aggressive immunosuppressive induction therapy with intravenous methylprednisolone 1 g daily for three days, followed by oral prednisolone at a dose of 1 mg/kg/day. This was combined with intravenous cyclophosphamide, administered as monthly pulses, in accordance with established lupus nephritis treatment protocols. Hydroxychloroquine 400 mg daily was introduced for long-term disease control, while cardiology input guided the management of pericarditis with nonsteroidal anti-inflammatory therapy, careful hemodynamic monitoring, and preparedness for pericardiocentesis should tamponade develop. Supportive measures included calcium and vitamin D supplementation for bone protection during corticosteroid therapy, pneumocystis prophylaxis with trimethoprim-sulfamethoxazole, vaccination updates, and strict counseling regarding sun protection and infection precautions.

During hospitalization, the patient demonstrated gradual clinical improvement, with resolution of fever, reduction in joint pain, and improved exercise tolerance. Serial monitoring of laboratory parameters confirmed a favorable response to therapy. After approximately three weeks, her renal function stabilized, with serum creatinine decreasing to 1.7 mg/dL and 24-hour proteinuria reducing to 2.9 g. Complement levels showed partial recovery, with C3 increasing to 55 mg/dL and C4 to 12 mg/dL, while anti-dsDNA antibody titers, although still elevated, showed a declining trend. Hemoglobin improved to 13.8 g/dL, and inflammatory markers decreased significantly. The patient's cardiac symptoms subsided, and echocardiography on follow-up revealed no progression of pericardial effusion. The arachnoid cyst was reviewed by neurosurgery, and as it remained asymptomatic without significant mass effect, a conservative monitoring strategy was advised.

The patient was discharged in stable condition on a regimen of tapering corticosteroids, hydroxychloroquine, and scheduled cyclophosphamide infusions. She was instructed to follow up with the rheumatology and nephrology clinics every 2-4 weeks for continued disease monitoring, with a long-term goal of achieving sustained remission of nephritis and preventing recurrence of carditis. At her most recent follow-up, she reported significant improvement in quality of life, with the ability to resume daily activities, underscoring the effectiveness of early recognition and coordinated multidisciplinary management in severe SLE.

## Discussion

SLE is a heterogeneous autoimmune disease with protean manifestations, ranging from mild cutaneous symptoms to fulminant multi-organ involvement. While renal and cardiac complications are well-recognized individually, their concurrent presentation in the early course of the disease is distinctly uncommon and presents unique diagnostic and therapeutic challenges [4]. In this case, the patient developed Class IV lupus nephritis and lupus carditis almost simultaneously, highlighting the unpredictable nature of SLE and the importance of maintaining a high index of suspicion when multiple organ systems are affected. The unusually high diagnostic score of 37 points using the 2019 EULAR/ACR criteria underscored the severity of systemic involvement, which is rarely observed at the time of initial presentation.

Lupus nephritis remains one of the most serious complications of SLE, occurring in 40-70% of patients depending on ethnicity and follow-up duration, and is strongly associated with morbidity and mortality if untreated. Cardiac involvement, on the other hand, occurs in roughly 20-30% of cases but often manifests later in the disease course, most frequently as pericarditis [5]. The coexistence of nephritis and carditis at the point of diagnosis raises important considerations for clinicians, as it necessitates the rapid initiation of aggressive immunosuppressive therapy to prevent irreversible damage to both the renal and cardiovascular systems. In our patient, prompt recognition and initiation of combined corticosteroid and cyclophosphamide therapy resulted in measurable improvement in renal function and the resolution of cardiac inflammation, illustrating the critical value of early, targeted treatment.

An additional complexity in this case was the incidental discovery of an arachnoid cyst during the workup for persistent headaches. While arachnoid cysts are typically asymptomatic and unrelated to autoimmune pathology, their presence can easily confound clinical interpretation, particularly in patients with suspected neuropsychiatric lupus [6]. In this case, the patient's subsequent clinical improvement with immunosuppressive therapy suggests the headaches were more likely SLE-related rather than attributable to the incidental cyst. This underscores the importance of comprehensive diagnostic evaluation in patients with overlapping neurological symptoms while also highlighting the risk of over-attributing unrelated findings to lupus activity. In clinical practice, such incidental findings necessitate careful multidisciplinary review to ensure appropriate management without unnecessary intervention.

From an educational perspective, this case illustrates several critical insights. First, the application of standardized diagnostic criteria such as the 2019 EULAR/ACR system is invaluable in guiding accurate diagnosis in complex presentations, ensuring that atypical but severe cases are not overlooked [7,8]. Second, it emphasizes the importance of a multidisciplinary management strategy, as rheumatologists, nephrologists, cardiologists, and neurologists each contributed essential expertise in optimizing the patient's care. Third, the case highlights the necessity of balancing aggressive immunosuppressive therapy with appropriate supportive measures, including infection prophylaxis, bone protection, and patient education, all of which are pivotal in improving long-term outcomes. Finally, it reinforces the need for clinicians to critically evaluate each new finding, whether related or incidental, to avoid misdiagnosis and inappropriate treatment.

This case provides an important reminder that SLE can present with rare but severe multi-organ involvement and that early recognition, systematic diagnostic application, and coordinated multidisciplinary intervention are key to preventing irreversible morbidity. It also highlights the clinical reasoning required to distinguish disease-related pathology from incidental findings, an essential skill for both trainees and experienced physicians managing complex autoimmune diseases.

## Conclusions

This case underscores the unpredictable and potentially life-threatening nature of SLE, particularly when multiple organ systems are affected simultaneously. The concurrent development of lupus nephritis and lupus carditis at initial presentation highlights the need for the immediate initiation of aggressive immunosuppressive therapy to prevent irreversible damage. Furthermore, the incidental finding of an arachnoid cyst illustrates that neuroimaging abnormalities should not delay or misdirect appropriate SLE treatment when clinical and laboratory features are compelling. Finally, the exceptionally high EULAR/ACR classification score of 37 points emphasizes the importance of multidisciplinary care coordination in patients with severe multi-organ involvement, ensuring timely intervention and improved long-term outcomes.

## References

[1] Dai X, Fan Y, Zhao X (2025). Systemic lupus erythematosus: updated insights on the pathogenesis, diagnosis, prevention and therapeutics. Signal Transduct Target Ther.

[2] Pryor KP, Barbhaiya M, Costenbader KH, Feldman CH (2021). Disparities in lupus and lupus nephritis care and outcomes among US Medicaid beneficiaries. Rheum Dis Clin North Am.

[3] Gros C, Fogel O, Boudhabhay I (2023). Challenging diagnosis of renal failure associated with severe neurological symptoms in a patient with mixed connective tissue disease. J Scleroderma Relat Disord.

[4] Justiz Vaillant AA, Goyal A, Varacallo MA (2023). Systemic lupus erythematosus. StatPearls.

[5] Bomback AS (2018). Nonproliferative forms of lupus nephritis: an overview. Rheum Dis Clin North Am.

[6] Wang JY, Hadi H, Arshad M, Whitney E (2025). A comprehensive review of arachnoid cysts. Cureus.

[7] Aringer M, Costenbader K, Daikh D (2019). 2019 European League Against Rheumatism/American College of Rheumatology classification criteria for systemic lupus erythematosus. Arthritis Rheumatol.

[8] Aslan E, Sahin S, Bektas S (2025). The performance of the 2019 EULAR/ACR classification criteria in childhood-onset systemic lupus erythematosus. Lupus.

